# Photo-Enhanced Peroxymonosulfate Activation via Well-Dispersed Cobalt Nanoparticles Encapsulated on Carbon Nitride for 2,8-Dichlorodibenzo-p-dioxin Removal

**DOI:** 10.3390/molecules30091917

**Published:** 2025-04-25

**Authors:** Yao Yue, Teer Wen, Yunfei He, Xuetong Qu, Jibo Dou, Yuchi Zhong, Jiafeng Ding, Hangjun Zhang

**Affiliations:** 1 School of Engineering, Hangzhou Normal University, Hangzhou 310018, China; 2023111010067@stu.hznu.edu.cn (Y.Y.); 2023111010099@stu.hznu.edu.cn (T.W.); heyyff@hznu.edu.cn (Y.H.); 2022111010069@stu.hznu.edu.cn (X.Q.); doujibo@hznu.edu.cn (J.D.); 2024111032004@stu.hznu.edu.cn (Y.Z.);; 2 Zhejiang Provincial Key Laboratory of Wetland Intelligent Monitoring and Ecological Restoration, Hangzhou Normal University, Hangzhou 311121, China

**Keywords:** 2,8-dichlorodibenzo-p-dioxin, peroxymonosulfate activation, cobalt-doped carbon nitride, reactive oxygen species, advanced oxidation processes

## Abstract

The removal of polychlorinated dibenzo-p-dioxins (PCDDs) via advanced oxidation processes (AOPs) poses a significant challenge due to their high toxicity and chemical stability. In this study, a series of well-dispersed cobalt nanoparticles supported on carbon nitrides (xCoCNs) was synthesized to activate peroxymonosulfate (PMS) for 2,8-dichlorodibenzo-p-dioxin (2,8-DCDD) degradation under visible light. The catalysts prepared were characterized using SEM, XPS, photoluminescence (PL), and UV-Vis diffuse reflectance spectroscopy (UV-Vis DRS). Among them, 2CoCN with an optimal Co content exhibited the highest photocatalytic efficiency, achieving 90.5% degradation of 2,8-DCDD within 160 min under visible light/persulfate oxidation (Vis+PMS+2CoCN system). Compared with other catalysts, 2CoCN exhibited superior optical performance and a narrower bandgap, enabling efficient excitation under visible light (Vis). Notably, all xCoCNs demonstrated pH adaptability, achieving complete degradation of 2,8-DCDD under neutral conditions (pH = 7) without additional acid/alkali adjustment. Through rigorous free radical capture experiments, it was demonstrated that SO_4_^•−^, ^•^OH and ^1^O_2_ were the primary reactive oxygen species (ROS) in the Vis+PMS+2CoCN system. The catalyst exhibited excellent reusability, with stable activity retained over five cycles. Based on these findings, degradation pathways and mechanisms of 2,8-DCDD in the 2CoCN+Vis+PMS system were proposed. This study presents an effective approach for PCDD abatement in wastewater treatment applications.

## 1. Introduction

Polychlorinated dibenzo-p-dioxins (PCDDs) have received extensive attention due to their adverse effects on the immune, neurological, endocrine, and reproductive systems in animals and humans [1]. Numerous studies have shown that PCDD pollution has spread globally. Dioxin-contaminated areas have been reported in many regions [2]. As a stable and persistent organic pollutant, a so-called “Poison of the Century”, PCDDs are transported from sources to the atmosphere, water, soils, and sediments, leading to severe environmental contamination [3]. Furthermore, these toxicants are continuously generated across various industries [4], such as waste incineration [5], organochlorine chemical production [6], and metallurgical processes [7]. There is an imperative need to develop efficient and innovative strategies for their removal.

A growing number of studies have focused on PCDDs removal, including chemical methods [8], biodegradation [9], photodegradation [10], activated carbon (AC) [11,12], and photocatalytic oxidation–reduction reactions [13,14]. For example, AC injection technology is the most common method for treating PCDDs from waste incineration, achieving over 95% removal efficiency when combined with bag filters [11,12]. However, AC application introduces additional carbon sources and necessitates secondary treatment, which increases the operational costs. Photocatalytic oxidation–reduction reactions have been widely applied for PCDDs’ degradation with promising results [13,14]. Advanced oxidation processes (AOPs) leverage highly reactive oxygen species (ROS) to degrade persistent organic pollutants. Among these, persulfate-based systems (e.g., PMS activation) have gained prominence due to their high redox potential (E0(SO_4_•^−^/SO_4_^2−^) = 2.5–3.1 VE0(SO_4_•^−^/SO_4_^2−^) = 2.5–3.1 V) and adaptability to diverse water matrices. Recent advances in catalyst design, such as single-atom metal sites and heterojunction engineering, have further enhanced AOP efficiency, as exemplified by innovative applications in dioxin degradation [15] and pharmaceutical removal [16]. Compared to traditional advanced oxidation processes (AOPs) relying on ^•^OH as the primary reactive oxygen species (ROS), emerging AOPs emphasize innovations in efficiency, resource conservation, and environmental sustainability, such as persulfate activation [17]. The removal of pollutants can be achieved through oxidation–reduction reactions between pollutants and sulfate radicals (SO^•^_4_^−^) activated by persulfate systems [18]. Zeng et al. [19] developed a persulfate activation method using Fe^2^⁺ and thermal energy for PCDDs treatment. Their results demonstrated that activated persulfates exhibited over 90% removal efficiency for organic contaminants under optimal conditions.

The integration of carbon materials with metal nanoparticles (NPs) has proven to be an effective strategy for activating persulfates. To enhance carbon material efficiency, empirical approaches such as reducing metal particle size and atomically dispersing them into a substrate have been developed [20]. Notably, cobalt (Co)-based catalysts exhibit superior performance in activating peroxymonosulfate (PMS) and generating reactive oxygen species (ROS) [21,22,23]. However, material engineering demands the immobilization of Co onto a suitable support to improve Co site dispersion, stability, and prevent particle aggregation. Graphitic carbon nitride (*g*-C_3_N_4_), a crystalline two-dimensional material, has emerged as a promising platform for water/wastewater treatment due to its structural robustness and low cost [24]. Previous studies have demonstrated the feasibility of using Co-based *g*-C_3_N_4_ catalysts for PMS activation, where *g*-C_3_N_4_ captures Co ions through Co-N interactions [25]. This interaction facilitates the formation of Co-Nx coordination complexes, which serve as an ideal substrate for stabilizing positively charged cobalt transition metal species [26]. Furthermore, *g*-C_3_N_4_ exhibits high photocatalytic efficiency under visible light. Therefore, we proposed using visible light to activate *g*-C_3_N_4_, generating electrons that accelerate the production of reactive oxygen species (ROS), thereby enhancing the removal efficiency of organic matter.

In this study, a series of well-dispersed Co-doped graphitic carbon nitride (xCoCN) composites were synthesized through a one-step method. Their performance in degrading 2,8-DCDD within visible light/persulfate (Vis+PMS+xCoCN) systems was comprehensively evaluated. The morphology, composition, optical properties, and stability of xCoCN were characterized. The effects of xCoCN dosage, persulfate concentration, pH, anions, and other parameters on 2,8-DCDD removal efficiency were systematically investigated using orthogonal experimental design. Free radical quenching and EPR analyses identified SO_4_^•−^, ^•^OH and ^1^O_2_ as the dominant reactive oxygen species (ROS) during degradation. Mechanistically, the synergistic visible light activation and persulfate oxidation pathways were elucidated. This study pioneers the integration of atomically dispersed Co-doped g-C_3_N_4_ with visible-light-driven PMS activation for PCDD degradation—a strategy unreported in prior studies. The synergistic design optimizes both ROS generation and catalyst stability, addressing key limitations in conventional AOPs.

## 2. Results and Discussion

### 2.1. Analysis of the Characteristics of xCoCNs

**Figure 1:**
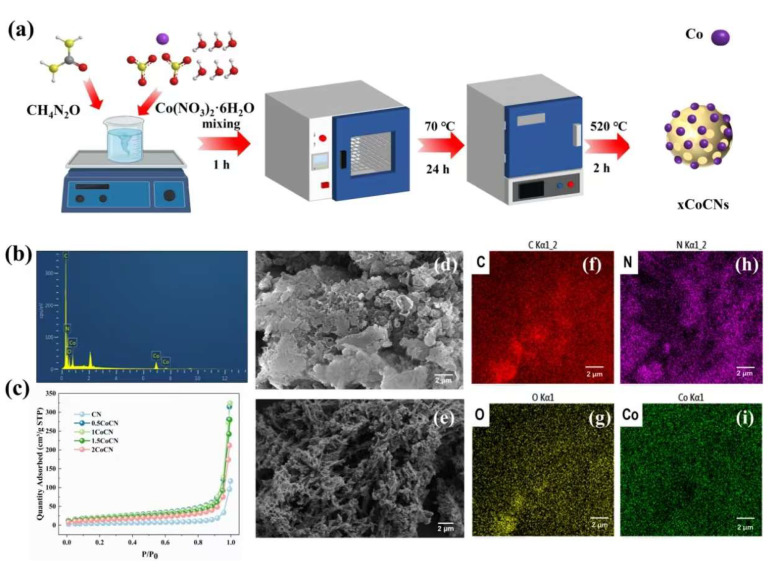
Schematic diagram for the synthesis of xCoCN (**a**); EDS spectra (**b**) and adsorption–desorption isotherms (**c**) of CN and xCoCN; SEM micrographs of CN (**d**), 2CoCN (**e**) and its corresponding elemental mappings: C (**f**), N (**g**), O (**h**), Co (**i**).

The synthesis process of xCoCN is illustrated in Figure 1a. Structural characterization begins with SEM (Figure 1d,e), revealing morphological evolution from CN (smooth layers) to 2CoCN (porous fragments). Subsequent EDS (Figure 1b) and XPS confirm Co doping and electronic interactions. As shown in Figure 1b, the EDS spectrum confirms that 2CoCN primarily consists of C, N, O, and Co. Corresponding elemental mappings (C: Figure 1f, N: Figure 1g, O: Figure 1h, Co: Figure 1i) demonstrate the uniform dispersion of Co on the *g*-C_3_N_4_ framework. Nitrogen adsorption–desorption isotherms (Figure 1c) exhibited Type IV isotherms with H3-type hysteresis loops, characteristic of mesoporous materials [27]. In the low-pressure region (P/P_0_ < 0.1), physisorption contributions are minimal, while micropore filling dominates at higher pressures. The surface areas of CN, 0.5CoCN, 1CoCN, 1.5CoCN, and 2CoCN were 61.97 ± 3.1, 70.11 ± 3.5, 82.25 ± 4.1, 86.66 ± 4.3, and 116.13 ± 5.8 m^2^·g^−1^, respectively (triplicate measurements). The progressive increase in surface area with Co loading correlates with an enhanced PMS adsorption capacity and active site accessibility; this trend underscores the critical role of nanostructural engineering in optimizing catalytic performance [26]. Scanning electron microscopy (SEM) images in Figure 1d,e reveal distinct morphological differences between pristine CN and 2CoCN. Pristine CN (Figure 1d) exhibits a compact, layered structure with smooth surfaces and minimal porosity. In contrast, 2CoCN (Figure 1e) displays a highly fragmented and porous architecture, characterized by interconnected nanoflakes with abundant voids (pore size range: 20–50 nm). This structural evolution is attributed to Co doping, which disrupts the graphitic stacking of g-C_3_N_4_ during calcination.

**Figure 2:**
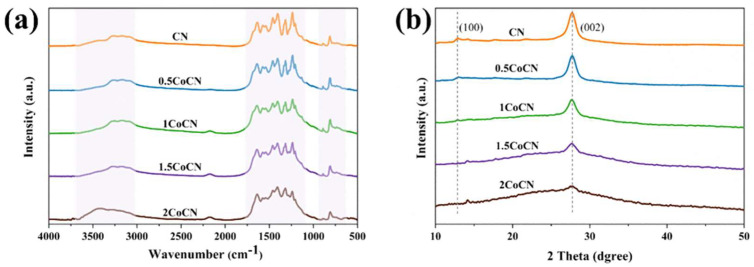
The ATR–FTIR spectra of CN and xCoCN (**a**), XRD pattern (**b**).

The FTIR spectra of the xCoCN samples are presented in Figure 2a. Characteristic absorption bands at 811 cm^−1^ (triazine ring) and 1240–1640 cm^−1^ (N-containing functional groups) confirm the *g*-C_3_N_4_ framework [28]. Notably, Co doping stabilizes the carbon nitride framework through electronic interactions, as evidenced by the retention of characteristic peaks at 811 cm^−1^ and 1240–1640 cm^−1^ despite variations in peak intensity due to Co-induced structural distortions [29]. The XRD patterns of pure CN and Co-doped samples (0.5CoCN, 1CoCN, 1.5CoCN, and 2CoCN) are shown in Figure 2b. Distinctive diffraction peaks at 27.5° (002) and 13.1° (100) correspond to the hexagonal phase of *g*-C_3_N_4_ (JCPDS No. 35-0664). The absence of additional peaks confirms the amorphous nature of Co species dispersion. Additionally, the absence of detectable metallic phases in all xCoCN samples indicates the atomic dispersion of Co within the carbon nitride frameworks. Furthermore, the intensity of XRD patterns decreased significantly upon incorporating Co(NO_3_)_2_·6H_2_O, which suggests an inverse relationship between cobalt loading and the crystallinity of triazine units.

**Figure 3:**
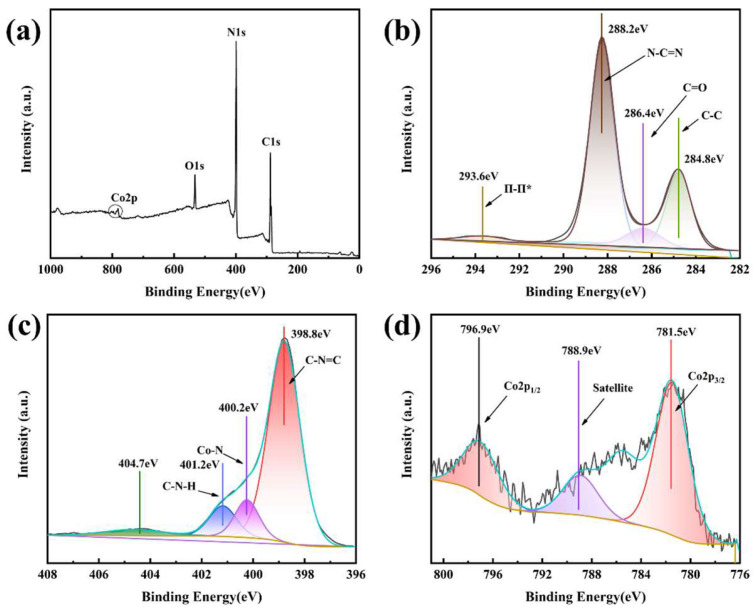
XPS spectra of 2CoCN full scan (**a**), C 1s (**b**), N 1s (**c**), and Co 2p (**d**).

XPS analysis was conducted to investigate the surface composition and chemical bonding configurations of CN and 2CoCN, with the results presented in Figure 3. Similar to the elemental mappings shown in Figure 1f–i, the survey spectrum of 2CoCN (Figure 3a) confirms the presence of C1s, N1s, O1s, and Co 2p signals. The C1s spectrum (Figure 3b) exhibits two dominant peaks at 284.8 eV (C-C bonds in sp^2^-hybridized graphitic carbon, a type of π-π* transition) and 288.2 eV (C=N-C bonds in triazine rings) [30]. High-resolution N1s (Figure 3c) resolves two peaks at 398.8 eV (pyridinic N) and 401.2 eV (pyrrolic N), accounting for 65% and 35% of the total N species, respectively [31]. For the Co 2p spectrum (Figure 3d), the primary peaks at 781.5 eV (Co 2p3/2) and 796.9 eV (Co 2p1/2) exhibit a splitting energy of 15.4 eV, characteristic of Co^2^⁺ species coordinated to N atoms in the carbon nitride framework, thereby confirming the successful integration of cobalt doping [32].

**Figure 4:**
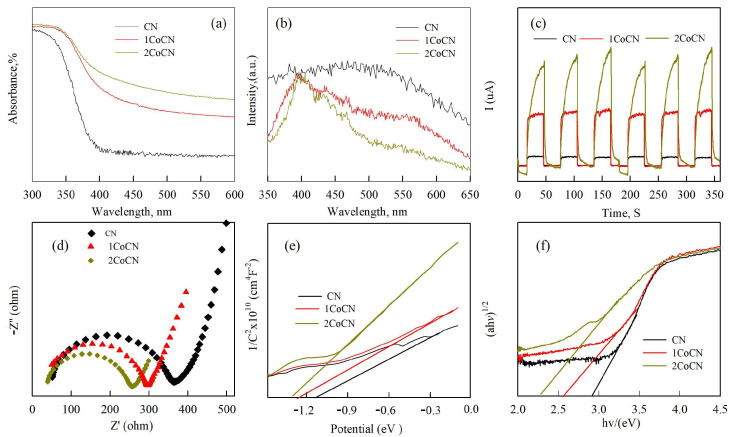
UV-vis DRS (**a**), PL (**b**) spectra, transient photocurrent (**c**), Nyquist plots of the electrochemical impedance spectrum (**d**), tauc curves (**e**) and estimated band structure (**f**) of CN and CoCN catalysts.

The diffuse reflection spectra of UV-Vis DRS, (Figure 4a) showed that the broad absorption intensity of 1CoCN and 2CoCN was significantly higher than that of pure CN, and that intensity increased with the increase in the Co content (λ onset = 480 nm vs. 450 nm). This indicates that the absorption capacity of visible light is improved [33]. The PL spectrum (excitation at 300 nm, Figure 4b) shows that the signal intensity is CN > 1CoCN > 2CoCN, in which the PL intensity of 2CoCN is reduced by 32%. The characteristic peak at 400 nm is present in all samples and is attributed to the trapping of photogenerated electrons by surface oxygen vacancies and defect states [34]. The broad PL peak centered at ~500 nm (attributed to defect-mediated recombination) shows reduced intensity with increasing Co content, confirming suppressed charge recombination. This improvement aligns with prior studies demonstrating that Co doping introduces defect states that trap photogenerated carriers, thereby minimizing radiative recombination pathways [34,35]. This improvement can be ascribed to the efficient transfer of photoelectrons from the CB of CN to the Co-doped CB in 2CoCN, which creates a built-in electric field to facilitate e^−^-*h*^+^ separation [36].

The transient photocurrent response of CN and 2CoCN under visible light illumination is shown in Figure 4c. The photocurrent density of 2CoCN rapidly increases to a steady-state value of 12 μA/cm^2^ within 1.2 s (90% signal rise time) upon light irradiation and decays to baseline within 2.5 s (90% signal recovery time) after switching off the light. In contrast, pristine CN exhibits a slower response (3.8 s rise time) and incomplete recovery due to severe charge recombination. The rapid response and recovery kinetics of 2CoCN confirm its superior charge separation efficiency and suppressed recombination, consistent with its enhanced photocatalytic activity [36]. As shown in Figure 4c, the responsiveness of CN showed the lowest response owing to the rapid complexation of photo-generated e^−^ and *h*^+^. When CN is combined with Co, the photoresponse capacity is significantly increased. The EIS spectra of CN and 2CoCN are shown in Figure 4d. It was discovered that pure CN had the big arc radius, suggesting that the material has a high recombination rate. Furthermore, the arc radius of 2CoCN has a slightly smaller variation, suggesting an improvement in charge separation efficiency. The findings show that 2CoCN has a higher effective photocatalytic potential than other modified nanomaterials.

The absorption band edge and the band gaps of the photocatalysts are calculated by the equation of E_g_ = 1240/λ_g_, where λ_g_ is the optical absorption edge of the semiconductor. The specific results demonstrate that E_g_ decreases from 2.88 to 2.4 eV with the ratio of Co increasing from 1 to 2, as shown in Figure 4f. The E_g_ of CN is calculated as 2.88 eV. It is well known that the narrower band gap can lead to better photocatalytic behavior [37]. Moreover, the Mott–Schottky plot (Figure 4e) was used to determine the flat-band potential (E_fb_) of pristine CN, 1CoCN, and 2CoCN as −1.08, −1.27, and −1.32 V (vs. NHE), respectively. Assuming E_fb_ approximates the conduction band potential (E_CB_) for n-type semiconductors [37], the E_CB_ values are −1.18, −1.37, and −1.42 eV (vs. NHE). The valence band potential (E_VB_) was calculated using the following equation:

E_VB_ = E_g_ + E_CB_

where Eg is the bandgap energy derived from UV-Vis DRS (Figure 4f). The calculated E_VB_ values for CN, 1CoCN, and 2CoCN are 1.70, 1.63, and 1.60 eV (vs. NHE), respectively. These results align with the enhanced photocatalytic activity of 2CoCN under visible light, as its narrower bandgap and lower E_VB_ facilitate efficient hole generation for ROS production.

### 2.2. Removal of 2,8-DCDD in the Vis+PMS+2CoCN System

**Figure 5:**
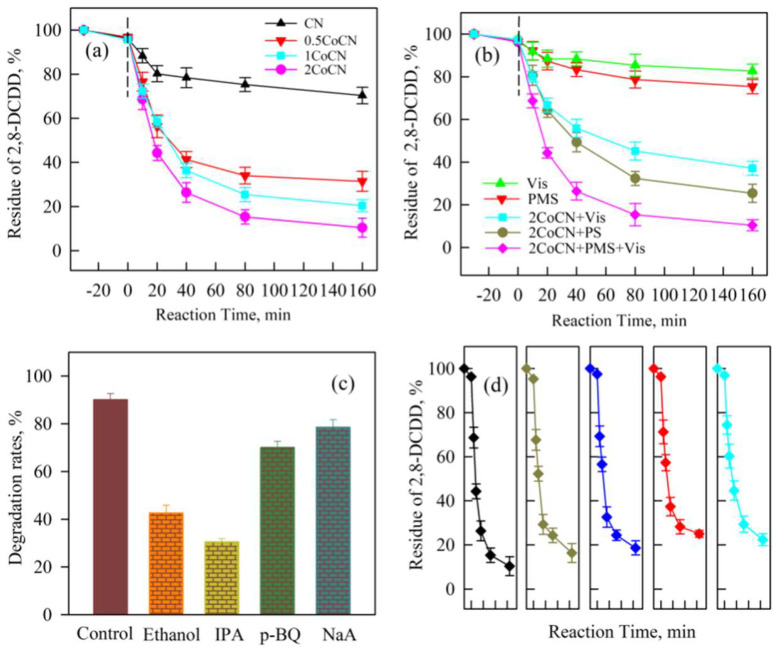
Removal of 2,8-DCDD by CN, 0.5CoCN, 1CoCN, and 2CoCN (**a**); removal of 2,8-DCDD in different reaction conditions (**b**); active species trapping experiments (**c**); the reusability of photocatalyst in the 2CoCN+PMS+Vis system (**d**).

The degradation performance of 2,8-dichlorodibenzo-p-dioxin (2,8-DCDD) was systematically evaluated in the Vis+PMS+2CoCN system, with the results presented in Figure 5. As shown in Figure 5a, residual 2,8-DCDD percentages were monitored over 160 min for catalysts CN, 0.5CoCN, 1CoCN, and 2CoCN. Notably, the 2CoCN catalyst exhibited the highest activity, achieving 90.5% degradation efficiency within 160 min. This superior performance can be attributed to optimized cobalt loading, which not only enhances peroxymonosulfate (PMS) activation but also facilitates reactive oxygen species (ROS) generation [38,39]. The comparative analysis of different systems, including Vis alone, PMS alone, Vis+2CoCN, PMS+2CoCN, and Vis+PMS+2CoCN, was presented in Figure 5b. It revealed that the Vis+PMS+2CoCN system achieved the highest degradation efficiency of 2,8-DCDD. Specifically, the residual percentage of 2,8-DCDD decreased to approximately 15% within 100 min, demonstrating a synergistic effect that arises from visible light irradiation, PMS activation, and the cobalt-based catalyst. This synergy is likely due to the enhanced generation of SO_4_^•−^, ^•^OH and ^1^O_2_, which efficiently break down persistent organic pollutants [39].

As illustrated in Figure 5c, scavenger experiments targeting reactive oxygen species (ROS) and hole trapping were conducted to identify the key ROS involved in the degradation pathway. The addition of scavengers—ethanol, isopropanol (IPA), p-benzoquinone (p-BQ), and sodium azide (NaA)—significantly inhibited 2,8-DCDD degradation. After quenching with IPA (36%), ethanol (42%), p-BQ (72%), and NaA (78%), the residual percentages of 2,8-DCDD decreased accordingly. These results indicate that SO_4_^•−^, ^•^OH and ^1^O_2_ collectively contribute to the degradation process, with SO_4_^•−^ and ^•^OH dominating due to their higher selectivity and longer lifetime [40]. The Vis+PMS+2CoCN system demonstrates exceptional potential for the efficient removal of 2,8-DCDD, leveraging the synergistic effects of visible light, PMS activation, and cobalt-based catalysis [41]. The potential applicability of 2CoCN depends on its reusability, a crucial parameter evaluated through multiple cycles. The experimental results show that the 2,8-DCDD removal efficiency did not significantly decrease after five consecutive cycles of recycling and reuse (Figure 5d), indicating that 2CoCN maintains good reusability.

**Figure 6:**
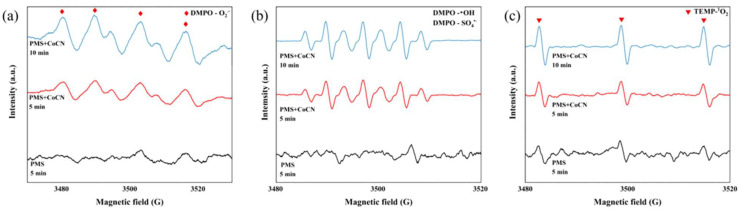
EPR spectra of the DMPO-^•^O_2_^−^ (**a**), DMPO-^•^OH and DMPO-SO_4_^•−^ adducts (**b**), TMPO-^1^O_2_ (**c**) recorded with the 2CoCN catalyst system under visible light irradiation using DMPO (10 mM) or TMPO (10 mM) as a spin trapping agent at 20 min.

In order to further clarify reactive oxygen species (ROS) generation, EPR spectroscopy was performed to identify reactive oxygen species (ROS) generated in the Vis+PMS+2CoCN system. As reported in our previous study [42], characteristic signals for SO_4_^•−^, ^•^OH, and ^1^O_2_ were detected (Figure 6), confirming their dominant roles in 2,8-DCDD degradation. Detailed spin-trapping protocols and spectral assignments are provided in [42]. The 2CoCN+PMS+Vis system demonstrates exceptional potential for 2,8-DCDD removal, capitalizing on synergistic effects of visible light, PMS activation, and cobalt.

### 2.3. Effects of Different Reaction Parameters

**Figure 7:**
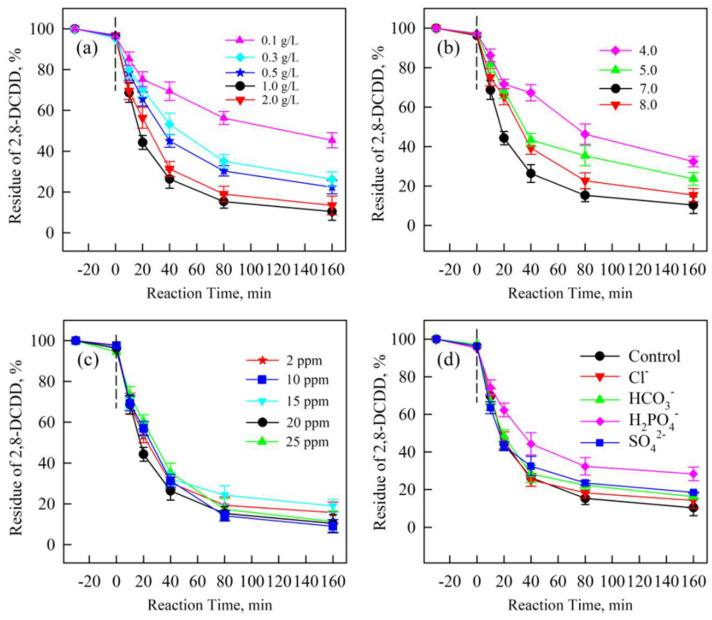
Effects of catalyst dosages (**a**), initial pH (**b**), initial 2,8-DCDD concentrations (**c**) and different anion concentrations (**d**) on 2,8-DCDD photocatalytic degradation rate in the 2CoCN+PMS+Vis system. Error bars indicate standard error.

As shown in Figure 7a, the degradation efficiency of 2,8-DCDD significantly increased with increasing the catalyst dosage from 0.1 g/L to 2.0 g/L. At 2.0 g/L, approximately 95% degradation was achieved within 160 min, suggesting that higher catalyst concentrations enhance the generation of reactive oxygen species (ROS), such as sulfate SO_4_^•−^, ^•^OH and ^1^O_2_, which are critical for 2,8-DCDD breakdown. This trend aligns with prior research showing that increased catalyst loading promotes PMS activation under visible light irradiation [43]. Figure 7b illustrates the effect of initial pH on the degradation efficiency of 2,8-DCDD. The optimal pH was determined to be 7.0, with degradation efficiency decreasing under both acidic (pH 4.0) and alkaline (pH 8.0) conditions. At neutral pH, the generation and persistence of SO_4_^•−^, ^•^OH and ^1^O_2_ are optimized. This is due to extreme pH conditions potentially scavenging these radicals or promoting the formation of less reactive species [44].

The degradation efficiency decreased with increasing initial 2,8-DCDD concentration, as shown in Figure 7c. The removal activity recorded a slight decline with the increase in initial concentration. It could be primarily attributed to the opacity of the solution being invariably enhanced to prevent photodegradation. Moreover, more active sites were filled by photocatalysts, which led to a decrease in SO_4_^•−^, ^•^OH and ^1^O_2_ generation, and then showed a decrease in its efficiency. This inverse relationship between degradation efficiency and initial pollutant concentration is consistent with prior studies on PMS-based systems. The presence of anions, particularly HCO_3_^−^ and HPO_4_^2−^, significantly suppressed the degradation of 2,8-DCDD, as shown in Figure 7d. This inhibition can be attributed to the scavenging of SO_4_^•−^, ^•^OH and ^1^O_2_ by these anions, which compete with 2,8-DCDD for the available ROS. Notably, HPO_4_^2−^ exhibited weaker inhibition compared to HCO_3_^−^, likely due to its lower affinity for capturing radicals. These findings align with prior research demonstrating the inhibitory roles of common anions in advanced oxidation processes (AOPs) [45].

**Figure 8:**
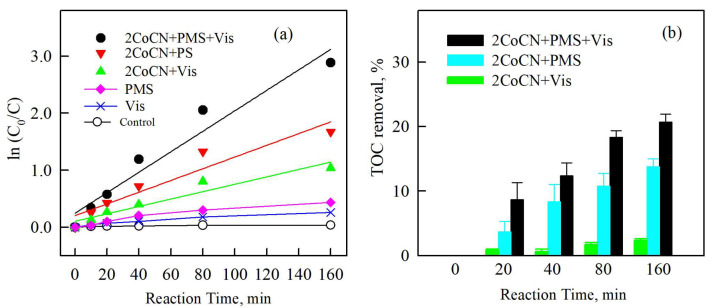
The pseudo-first-order rate constant of 2,8-DCDD removal by CN, 0.5CoCN, 1CoCN, and 2CoCN in the 2CoCN+PMS+Vis system (**a**). Mineralization efficiency of 2,8-DCDD in the Vis+PMS+2CoCN system (**b**).

The first-order kinetic reaction model of different systems to the dechlorination of 2,8-DCDD is shown in Figure 8a. The first-order kinetic reaction model was also improved in the same order: ln (C/C0) = −kt. And the Half-lives (t_1/2_) were calculated by t_1/2_ = ln2/k. Obviously, the catalytic efficiency enhanced with the addition of PMS and Vis, and the corresponding k values increased from 0.011 h^−1^ to 0.44 h^−1^. This significant improvement is due to a higher decrease in SO_4_^•−^, ^•^OH and ^1^O_2_ generation. As shown in Figure 8b, TOC tests showed that more than 20% of the pollutant can be completely degraded within 160 min, proving that Vis+PMS+2CoCN system has a certain ability to mineralize 2,8-DCDD, but this is lower than the removal efficiency of 2,8-DCDD. This is mostly due to the intermediates created by 2,8-DCDD degrading into CO_2_ and other inorganic chemicals at a substantially slower rate than 2,8-DCDD itself.

### 2.4. Photocatalytic Mechanism of xCoCNs

**Figure 9:**
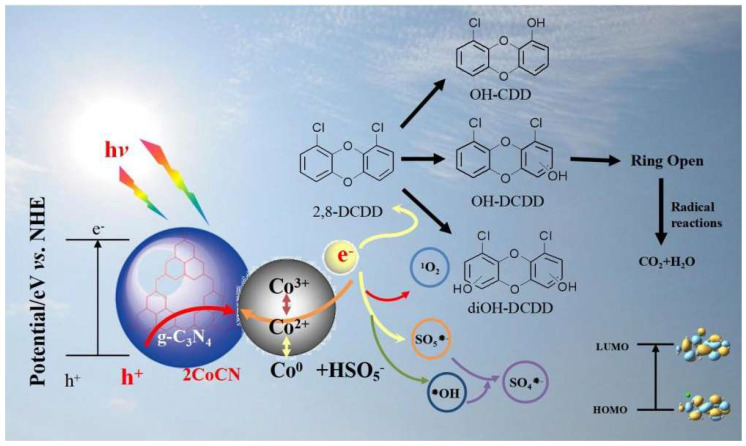
Proposed degradation mechanisms in the Vis+PMS+2CoCN system.

Based on radical quenching tests, electrochemical measurements, XPS analysis, and EPR spectroscopy, we proposed the degradation mechanism of 2,8-DCDD in the 2CoCN+PMS+Vis system (Figure 9). According to these results and previous studies [46,47], the generation process of reactive oxygen species (ROS) proceeded as follows: Surface Co(II) reacted with HSO₅^−^ to produce SO_4_^•−^, ^•^OH and ^1^O_2_. To identify intermediates in the 2,8-DCDD oxidation process, gaseous intermediates were analyzed by GC–MS, while liquid intermediates were characterized using HPLC. Based on these data, the proposed degradation pathways of 2,8-DCDD are illustrated in Figure 8. For example, hydroxylated intermediates such as OH-DCDD and diOH-DCDD were detected in the Vis+PMS+2CoCN system. These intermediates were further oxidized and cleaved into smaller fragments, culminating in chemical destabilization and eventual mineralization to CO_2_ and H_2_O.

## 3. Materials and Methods

### 3.1. Materials

Urea COH_4_N_2_, purity >97%, and cobalt nitrate hexahydrate Co(NO_3_)_2_·6H_2_O, purity >95%, were purchased from Aladdin Reagent (Shanghai, China). Potassium monopersulfate triple salt (42–46% KHSO_5_ basis) and L-histidine (purity ≥99%) were obtained from Macklin (Shanghai, China). 2,8-Dichlorodibenzo-p-dioxin (2,8-DCDD, purity >99%) standards and their derivatives were purchased from Accustandard (New Haven, CT, USA). HPLC-grade solvents, including isopropanol (≥99.7%), toluene (≥99%), n-hexane (≥99%), anhydrous ethanol (≥99.7%), methanol (≥99.7%), and acetylacetone (≥99%), were purchased from Sinopharm Chemical Reagent Co., Ltd. (SCR, Shanghai, China). Ultrapure water (18.2 MΩ·cm resistivity) was obtained by purification using a Direct-Q 3UV system (Merck, Boston, MA, USA).

### 3.2. Synthesis of Catalysts

The xCoCN catalysts were synthesized via a one-step method (Figure 1a). Typically, predetermined amounts of Co(NO_3_)_2_·6H_2_O (0.5, 1.0, 1.5, 2.0 mmol) and urea (10 g) were dissolved in deionized water. The mixture was stirred continuously for 1 h to ensure complete hydrolysis. After filtration, the resulting slurry was ultrasonicated for 1 h, followed by drying at 70 °C for 24 h. Finally, the samples were calcined at 520 °C for 2 h with a heating rate of 5 °C/min. The resulting powders were filtered, washed with deionized water, and dried overnight at 60 °C.

### 3.3. Characterization

The X-ray diffraction (XRD) analysis of CoCN-x catalysts was performed on an X’ pert Pro diffractometer (XRD-6000; Shimadzu, Kyoto, Japan) using Cu-Kα radiation (λ = 0.1546 nm). Fourier-transform infrared spectroscopy (FTIR) measurements were conducted on a Thermo Scientific Nicolet iS20 instrument (Thermo, Waltham, MA, USA). X-ray photoelectron spectroscopy (XPS) was carried out using a Thermo Fisher Scientific Escalab 250 instrument (Thermo, Waltham, MA, USA). BET surface area analysis of the composite was performed using a 3H-2000PS2 apparatus (Beishide, Shanghai, China) with N_2_ adsorption–desorption isotherms recorded at 77 K. Photoluminescence spectra (PL) were recorded using a Horiba Fluorolog-3 tau fluorescence spectrophotometer (Tokyo, Japan). Electrochemical analysis (i-t curves and EIS) was performed using a CHI 650D electrochemica (Shanghai, China) with a three-electrode configuration. UV-Vis absorption and diffuse reflectance spectroscopy (UV-Vis DRS) spectra were obtained using a TU-1901 spectrophotometer (Pgeneral, Beijing, China). EPR measurements were conducted following the methodology described in [42]. Scanning electron microscopy (SEM) images were acquired with a Zeiss Sigma 300 microscope (Carl Zeiss AG, Baden-Württemberg, Germany) at 2.0 kV. Elemental mapping and EDS analysis were performed using a Zeiss Sigma 300 SEM equipped with an Oxford X-Max 80 detector (Abingdon, UK), operated at 15 kV with a 10 mm working distance.

### 3.4. Degradation Setup of 2,8-DCDD

Batch experiments were conducted in a jacketed quartz photoreactor with a top-illuminated configuration. The reaction was performed under visible light (420 nm filter) using a 300 W Xe lamp (Ushio, Tokyo, Japan). In each experiment, 0.1 g of the catalyst was added to 200 mL of the 2,8-DCDD solution (50 μg/L) and stirred magnetically at 200 rpm for 60 min in darkness to establish adsorption–desorption equilibrium (resulting in a catalyst concentration of 1.0 g/L). Subsequently, the Xe lamp was turned on, and 20 mM PMS solution was simultaneously introduced. The pH of the reaction system under air-saturated conditions was maintained at 7.0. At regular intervals, 1 mL subsamples were withdrawn, extracted twice with toluene/n-hexane (*v*/*v* = 1:1, 2 mL each time), shaken horizontally for 10 min, and centrifuged at 5000 rpm for 10 min. The supernatants were collected and analyzed by gas chromatography–mass spectrometry (GC-MS). The pH range from 3.0 to 9.0 was adjusted using 0.1 M H_2_SO_4_ or 0.1 M NaOH solutions. In the reactive oxygen species (ROS) scavenging experiment, terephthalic acid (TBA), methanol (MeOH), 1,4-benzoquinone (p-BQ), and sodium azide (NaA) were used as scavengers for ^•^OH, ^•^O_2_^−^, SO_4_^•−^ and ^1^O_2_, respectively. The reusability of the catalyst was evaluated through five consecutive cycles. After each reaction, the catalyst was filtered using a 0.45 μm filter and washed three times with toluene/n-hexane (*v*/*v* = 1:1) and distilled water to remove residual 2,8-DCDD and products, followed by drying for reuse.

### 3.5. Chemical Analysis

The concentrations of 2,8-DCDD, 2-CDD, and DD in the reaction samples were quantified using a GC-MS system (Agilent 7890A GC coupled with an Agilent 5975C MS, Agilent Technologies, Wilmington, DE, USA) operated in electron impact (EI) ionization mode with full-scan acquisition (*m*/*z* 50–500). A BD-1 column (0.25 mm i.d. × 30 m, Agilent) was employed. The GC injector, ion source, MS detector, and transfer line temperatures were maintained at 300 °C, 250 °C, 180 °C, and 270 °C, respectively. Helium was used as the carrier gas at a constant flow rate of 1.0 mL/min. The residual concentrations of target compounds were determined based on their characteristic ions (*m*/*z* 252/217 for 2,8-DCDD; 220/218 for 2-CDD; 186/184 for DD), which were validated using authentic standards.

## 4. Conclusions

This study investigated the enhanced photocatalytic degradation of 2,8-DCDD using novel Co-doped carbon nitride (xCoCNs) catalysts in a visible light (Vis) + peroxymonosulfate (PMS) system. The xCoCNs exhibited superior photoactivity compared to pristine CN. Under optimal conditions (catalyst loading = 0.1 g/L, pH = 7.0, vis+PMS+2CoCN system for 60 min), 90.5% degradation efficiency of 2,8-DCDD was achieved within 160 min. The enhanced performance was attributed to the expanded light absorption range into the visible spectrum due to Co doping and the suppression of e^−^-*h*^+^ recombination. The addition of PMS significantly improved the quantum yield of photoexcited electrons through SO_4_^•−^, ^•^OH and ^1^O_2_ generation. ESR analysis confirmed that the primary reactive oxygen species (ROS) involved were SO_4_^•−^, ^•^OH and ^•^O_2_^−^. The catalyst maintained reasonable activity and structural stability after five consecutive cycles. Based on identified intermediates and products, plausible degradation pathways and mechanisms for 2,8-DCDD in the xCoCN+Vis+PMS system were proposed. Overall, this study demonstrated a novel photocatalyst with nano structural advantages, which showed excellent potential for the remediation of 2,8-DCDD in wastewater treatment applications. While the 2CoCN catalyst exhibits promising performance under controlled laboratory conditions, several challenges must be addressed for practical implementation:

Scalability: the one-step synthesis of xCoCN requires precise control of calcination parameters (e.g., temperature ramping), which may pose challenges in large-scale production.

Real-water complexity: the current study uses synthetic wastewater; the presence of natural organic matter (NOM) or suspended solids in real wastewater could scavenge ROS or block active sites, necessitating pre-treatment steps.

In addition, future work will focus on:

Hybrid systems: integrating 2CoCN with membrane filtration or bioaugmentation to enhance mineralization efficiency and reduce energy consumption.

Mechanistic deepening: employing in situ spectroscopic techniques (e.g., operando XAS) to probe Co speciation during PMS activation.

## Data Availability

All data supporting the results can be found within the manuscript.
